# In Vitro effect of the ethanolic extract of *Tephrosia Vogelii* on *Rhipicephalus Sanguineus* in Abomey-Calavi 

**Published:** 2015

**Authors:** Dougnon Tossou Jacques, Adéhan Safiou, Houessionon Jédirfort, Farougou Souaïbou

**Affiliations:** *Research Laboratory in AppliedBiology, Polytechnic School of Abomey-Calavi, University of Abomey-Calavi, 01BP2009 Abomey-Calavi, Republic of Benin*

**Keywords:** *Dog*, *Tephrosia vogelii*, *Rhipicephalus sanguineus*, *Abomey-Calavi*

## Abstract

**Objectives:**

Ticks are vectors of several diseases, of which many are zoonosis transmissible to humans. The use of *Tephrosia* leafs’ extract as a low cost acaricide is spreading among farmers in central Kenya.

**Materials and Methods:**

The present study’s aim is to inventory endogenous control methods against dogs’ ticks among which *Rhipicephalus sanguineus*, in the Municipality of Abomey-Calavi. From September to October 2013, a survey was made on forty randomly selected breeders and ticks samples were collected on forty dogs. The web platform, www.epicollect.net, was used for the survey. In total, 77.5% (n=40) of examined dogs were infested with ticks

**Results:**

Three species of ticks were identified: *Rhipicephalus sanguineus*, *Haemaphysalis leachi,* and *Amblyomma variegatum*. They were found on 77.5%, 17.5%, and 15% of examined dogs, respectively. The numerical abundance of the three species was 87.06%, 11.9%, and 1.03%, respectively. The average number of ticks per animal was 16.83±5.04, 2.3±1.64, and 0.2±0.08 for *Rhipicephalus sanguineus, Haemaphysalis leachi, *and* Amblyomma variegatum, *respectively. Farmers used manual diptank (67.5%), plant ash (37.5%), petroleum (12.5%), motor oil (2.50 %), and sea water (7.5%) to fight against ticks.

**Conclusion:**

The phytochemical screening of the leafy stem’s powder of *Tephrosia vogelii* revealed the presence of catechol tannins, saponins, sugars, leuco-anthocyanins, polyterpenes, and sterols. A 100% larval mortality was observed at the concentration of 20% the ethanolic extract of the leafy stem of *Tephrosia vogelii*. The LC_50_ of this ethanolic extract against *Rhipicephalus sanguineus* larvae was equal to 2.6%.

## Introduction

Farming is heavily involved in the development of human societies by reducing unemployment and improving living conditions of farmers and populations (Kamuanga, 2003 ▶). 

However, breeding of pets including dogs, is often overlooked mainly in developing countries such as Benin (Hughes and Macdonald, 2013 ▶). Therefore, data on the importance and impact of social integration of these species are rare and not updated in the West African area, particularly in Benin. However, the dog is the most bred carnivore in the world (Wandeler et al., 1993 ▶). In Africa, population of dogs is currently estimated at 87.6 million heads (Hughes and Macdonald, 2013 ▶). Dogs play various roles including guarding home, hunting in rural areas, and narcotics detection (Smith et al., 2000 ▶). This therefore causes it to be in permanent contact with humans in both urban and rural environments.

Nevertheless, due to several social factors including low economic income and low level of education of the population, diseases of dogs are often ignored and little known in Benin. Yet, more than sixty zoonoses are associated to dog (Matter and Daniels, 2000 ▶). It is therefore important to study canine diseases and their biological vectors. These vectors are primarily insect larvae, fleas, lice, and above all, ticks (Colley, 2011 ▶). Indeed, in addition to mechanical stress and spoiling action during infestation, ticks are responsible for many diseases including zoonoses that are important in both dogs and their owners or vets (Estrada-Pena, 2005 ▶). Several cases of human infestation by dog’s ticks were already reported (Dworkin et al., 1999 ▶; Parola and Raoult, 2001 ▶).

A second important aspect to consider in the study of canine diseases is the development of alternatives methods to fight against these diseases vectors. Indeed, the conventional use of synthetic molecules to control ticks is causing many problems among which the emergence of resistance in tick populations, environment pollution, and the high cost of synthetic acaricides can be mentioned (Morel and Troncy, 2000 ▶; Estrada-Pena, 2005 ▶).

This study aims to identify endogenous control methods used against dogs’ hard ticks in the Municipality of Abomey-Calavi in southern of Benin. For that, various ticks on dogs were identified and their prevalence and abundance assessed. Moreover, traditional control methods used against dog’s ticks in the Municipality of Abomey-Calavi were recorded and acaricidal potential of the ethanolic extract of leafy stem of *Tephrosia vogelii *evaluated.

## Materials and Methods

Study environment

The present study was conducted in the Municipality of Abomey-Calavi. It is located south of the Republic of Benin in the Department of Atlantic. Abomey-Calavi is between 6° 26' 55'' North and 2° 21' 20'' East at 55 m altitude. It is bordered on the North by the Municipality of Zè, on South by the Atlantic Ocean, on the East by the Municipalities of Sô- Ava and Cotonou, and on the West by the municipalities of Tori-Bossito and Ouidah. Abomey-Calavi is the largest Municipality of the Atlantic Department (over 20%) and occupies an area of 650 km² or about 0.6% of the national area. Climate is sub-equatorial characterized by four seasons with two rainy and two dry. Rainfall is relatively high; they average 100 mm of rain per month, or 1200 mm per year. The humidity is high throughout the year with an average of 80%. The temperature remains high: 26.6±7 °C (Akoègninou, 2004 ▶).

**Figure 1:**
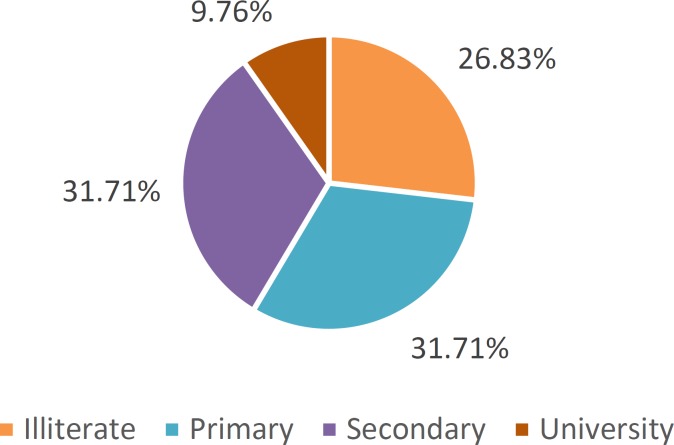
Education levels of dog breeders in the Municipality of Abomey-Calavi

The work of this study concerned thirty-four villages and/or cities of the nine districts of the commune. The localities were chosen randomly. Coordinates of sampling and survey sites are presented in Figure 1.

**Population sample and period of study**

Samples used in this study were gathered between September 2 and October 1, 2013 on forty randomly selected for four animals at least arrondissement dogs. All selected dogs followed two criteria: they were local race and did not get important and regular modern veterinary care (including synthetic acaricides). The selection of dog breeders was random and was not subjected to any social, economic, or professional criteria.

**Survey, sampling, and identification of ticks**

For data collection, a prevalence survey was conducted. The web platform www.epicollect.net, developed by Google Inc., was used as a part of the investigation. Epicollect is a web application that allows collecting epidemiological data based on questionnaires using a smartphone with GPS. The information collected in this study was related to geographical coordinates, dog owners (level of education and sex), the use of unconventional or traditional methods to control ticks, the vaccination status of dogs with regard to rabies, and the presence or absence of ticks on dogs.

Moreover, sampling of ticks by pulling was performed on all dogs. Most important samples sites were pinnae, neck, back, and interdigital spaces. However, the rest of the body was also examined. The engorged females and the male that were often attached to them were kept alive in sealed plastic bottles for a better identification based on the discriminative characteristics of males. The remaining samples were stored in 70% alcohol and brought to the laboratory for identification and counting. Small bottles were used to enclose ticks collected from animals. The sampling equipment consisted of tongs, bottles, and muzzles. At the end of sampling, a label with the necessary information (identification number, date and place of sampling, and sex of the animal) was introduced directly into each bottle before its closure.

The identification of ticks was made in the laboratory based on the Ticks of Domestic Animals in Africa: a Guide to Identification of Species (Walker et al., 2007 ▶). Gender and species, the evolutionary stage, and sex of each tick examined were recorded. Ticks were identified through binocular magnifier and microscope.

**Culture of** ***Rhipicephalus sanguineus*** **at laboratory**

Engorged females of *Rhipicephalus sanguineus* brought to laboratory were laid in dry bottles sealed with mosquito nets. They were then placed in a Memmert oven at 27±1 °C and a relative humidity between 85-90% for egg laying, hatching, and maturation of larvae hatched from the eggs. Preoviposition, oviposition, and incubation duration were determined by recording the dates of laying and hatching.

**Ethanolic extract and phytochemical screening of** ***Tephrosia vogelii***

Leafy stems of *Tephrosia vogelii* were dried at ambient laboratory temperature and finely powdered. The ethanolic extract was obtained by maceration of 100 g of powder in 500 ml of 96° ethanol in sealed glass vials for seven days. The extracted solution was subjected to evaporation under vacuum and low temperature (50 °C) in a Rotavapor R200 to let the alcohol evaporate. The resulting solution was placed in a Memmert oven at 50 °C to complete evaporation of the solvent.

To assess the presence of secondary metabolites including polyphenols, tannins, alkaloids, and anthocyanins contained in the leafy stem of *Tephrosia vogelii*, a phytochemical screening was performed on its powder in the Laboratory of Applied Research in Chemistry (LERCA) of the University of Abomey-Calavi (Benin).

**Efficiency test** ***in vitro***

To evaluate acaricidal activity of *Tephrosia vogelii*, larvae of *Rhipicephalus sanguineus* were subjected to the ethanolic extract of the leafy stems. The Larval Packet Test (LPT) was used and various concentrations were tested.

A mixture of two volumes of olive oil and one volume of trichloroethane was used as a control solution, and as solvent for the preparation of six solutions of concentrations 0.625%, 1.25%, 2.5%, 5.0%, 10%, and 20 % of the extract. For each concentration, two replicates were performed. Little paper packets, of dimensions 7.5×8.5 cm^2^, were made ​​with Whatman paper N° 1. The packets were impregnated with 0.67 ml of solution and were dried in ambient temperature for two hours to evaporate the solvent. After drying, the side edges of each packet were sealed with two clasps. Approximately 100 to 150 larvae, 7 to 14 days old were put in the packets. Afterwards, a third clasp was placed to close the top of each packet, to prevent larvae from escaping.

These larval packets were stored at ambient laboratory temperature for 24 hours. The packets were then opened and larvae were observed separately with eyes and stereoscope. Larvae were stimulated by blowing lightly on them and those that could not walk or move were considered dead. The larval mortality rate (R) was determined by the formula:

R=Dead larvaeTotal amount of larvae x 100

Statistical Analysis

*Proc GLM* of Statistical Analysis System (SAS, version 2006) were used for variance analysis of data collected during the survey. Frequencies of each tick species and their relative abundances were calculated. The percentages were compared pairwise using the Z test and means using the Student’s t-test. Data from *in vitro* assay were analyzed in PoloPlus (version 1.0) to determine the lethal doses as well as the confidence intervals and efficiency logarithmic curve expressing the dose-effect relation.

## Results

Population studied

The results of the survey show that female breeders represent only 17.5% of owners surveyed. The educational level of farmers ranged from uneducated to university level (Figure 1). Regarding dogs studied, they were all of local breed, 72.5% males. Vaccination against rabies within the sample was small (25%).

**Tick species**

**Figure 2:**
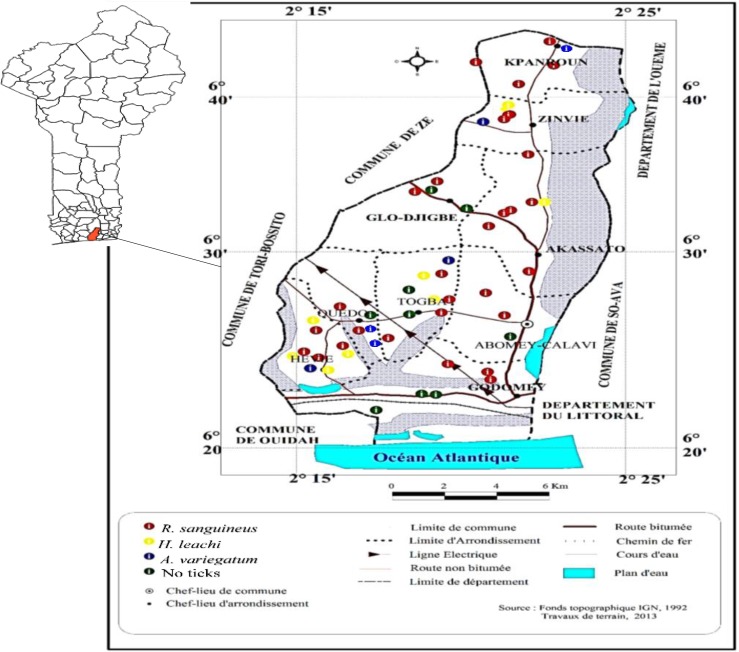
Distribution of hard ticks of the dog in the Town of Abomey-Calavi.

Three genus of hard ticks were identified: Rhipicephalus, Haemaphysalis, and Amblyomma. The brown dog tick, *Rhipicephalus sanguineus*, the yellow dog tick, *Haemaphysalis leachi*, and the tropical bont tick, *Amblyomma variegatum* represented these three genus, respectively. The distribution of all ticks found in the Commune of Abomey-Calavi is shown in Figure 2.

**Frequency and relative abundance of ticks**

**Figure 3:**
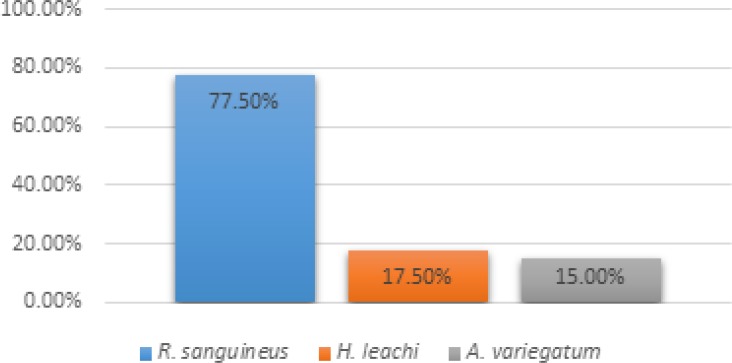
Prevalence of dogs’ ticks in the Municipality of Abomey-Calavi

This study revealed a high incidence of tick infestations in dogs. Indeed, 77.5% of the examined dogs were infested with ticks. Regarding tick species encountered, *Rhipicephalus sanguineus*,* Haemaphysalis leachi*, and *Amblyomma variegatum* were found on 77.5%, 17.5%, and 15% of dogs, respectively. There was a significant difference at 5% level between the frequency of infestation Rhipicephalus sanguineus on the one hand and those of a *Haemaphysalis leachi* and *Amblyomm variegatum *on other hand, but the last two were not significantly different (Figure 3).

Regarding the relative abundance of ticks (Tables 1, 2, and 3), 773 ticks were collected. *Rhipicephalus sanguineus* was the most abundant tick (87.06%), followed by *Haemaphysalis leachi* (11.9%) and *Amblyomna variegatum *(1.03%). The average number of ticks collected from each dog was 16.83±5.04, 2.3±1.64, and 0.2±0.08 for *Rhipicephalus sanguineus*, *Haemaphysalis leachi,* and *Amblyomma variegatum*, respectively. The numerical abundance of *Rhipicephalus sanguineus* was significantly different from those of the other two species of ticks (p<0.01). There was no significant difference in the numerical abundance of ticks (p>0.05) according to sex of dogs.

**Traditional control methods used against dogs’ ticks**

**Figure 4:**
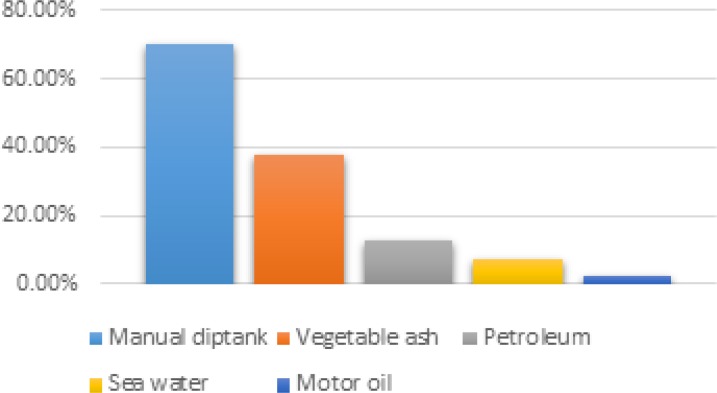
Utilization rate of control methods against ticks

**Figure 5:**
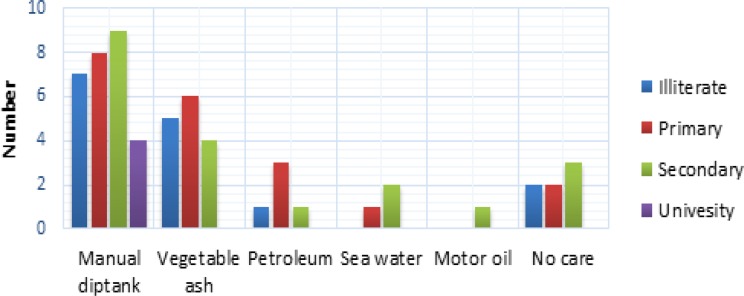
Traditional control methods by levels of education

The survey revealed that people use various alternative methods in the fight against ticks. 87.5% of dog breeders used at least one alternative method to fight against ticks. Besides the manual diptank used by 67.5% of dog’s breeder surveyed whatever their level of education, vegetable ash, petroleum, motor oil, and sea water were used by 37.5%, 12.5%, 2.50%, and 7.5% of breeders, respectively (Figures 4 and 5).

**Table 1:** Infestation depending on the species and life stage of the ticks.

**Ticks’ stage**	***R. sanguineus***		***H. leachi***		***A. variegatum***		**Significance**
	**Means**	**ET**	**Means**	**ET**	**Means**	**ET**	
**Female**	7.56^a^	1.52	0.6^b^	1.52	0^b^	1.52	
**Male**	8.86^a^	1.94	1.19^b^	1.94	0b	1.94	
**Nymph**	0.34^a^	0.09	0.04^a^	0.09	0.017^a^	0.09	NS
**Larva**	0^a^	0.016	0^a^	0.016	0.17^a^	0.016	NS
**Total**	16.83^a^	5.04	2.3^b^	1.64	0.2^b^	0.08	

Means in the same row followed by different letters are significantly different at 5% level;

**: p<0.01;

*** p<0.001; NS: p> 0.05; ET: Error Type.

**Table 2:** Relative abundance of ticks

**Ticks’ stage**	***R. sanguineus***	***H. leachi***	***A. variegatum***	**Significance**
**Female**	293^a^	30^b^	0^b^	
**Male**	365^a^	61^b^	0^b^	
**Nymph**	15^a^	1^a^	7^a^	NS
**Larva**	0^a^	0^a^	1^a^	NS
**Total**	673^a^	92^b^	8^b^	
**%**	87.06^a^	11.9^b^	1.03^b^	

Means in the same row followed by different letters are significantly different at 5% level;

**: p< 0.01;

*** p< 0.001; NS: p> 0.05; ET: Error Type.

**Table 3:** Numerical abundance of ticks in relation to sex of dogs

**Dogs’ sex**	***R. sanguineus***		***H. leachi***		***A. variegatum***		**Total**	
	Moyenne	ET	Moyenne	ET	Moyenne	ET	Moyenne	ET
**Female**	16.64	3.45	0.82	0.44	0.18	0.18	17.64	3.24
**Male**	16.90	6.88	2.86	2.26	0.21	0.09	19.97	8.84
**Significance**	NS		NS		NS		NS	

NS : p> 0.05 ; ET : Error Type

Many farmers used two or three of these methods. Sea water was used by the owners to shower the dog. Petroleum and motor oil were used by direct spraying on tick or on infested areas. Regarding the vegetable ash, breeders mention no particular vegetable species. They used vegetable ash by dispersion in dogs’ shelters. When the dog had no shelter, as was often the case in the villages, farmers willingly allowed the animal to lie down and sleep in the ashes of traditional kitchens. 

**Phytochemical screening of the ethanol extract of** ***Tephrosia vogelii***

The phytochemical screening of the powder of leafy stems of *Tephrosia vogelii* revealed the presence of several important secondary metabolites in veterinary pharmacopoeia for their biological action. Table 4 shows the chemical composition of the leafy stems of *Tephrosia vogelii*.

**Table 4:** Chemical composition of the leafy stems of T. vogelii

**Secondary metabolites **		**Leafy stem ** ***T. vogelii***
**Anthocyanins **		-
**Alkaloids **		-
**Free anthraquinones **		-
**Combined anthraquinones**	O-glycosides	-
	O-glycosides with reduced genin	-
	C-glycosides	-
**Flavonoids **		-
**Tannins **	Gallic	-
	Catechol	+
**Mucilages **		-
**Saponins **		+
**Reducing compounds **		+ / -
**Leuco-anthocyanin **		+
**Coumarins **		-
**Sterols and polyterpenes **		+

+: Presence; -: Absence; +/-: Traces

**Laboratory culture of** ***Rhipicephalus sanguineus***

From the sampling of engorged females of *R. sanguineus* to egg hatching, preoviposition, oviposition, and incubation durations were determined. Preoviposition lasted 5 days, oviposition 18 days and incubation 17 days.

**Efficiency test in vitro**

**Figure 6:**
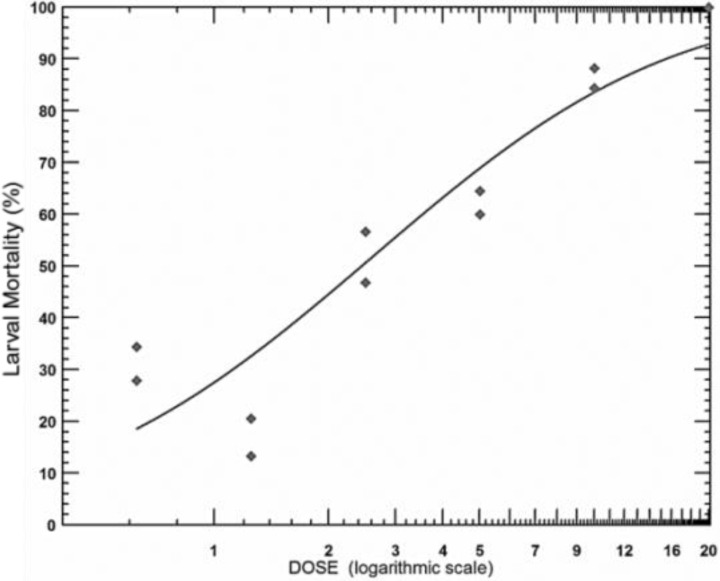
Larval mortality induced by ethanolic extract of Tephrosia vogelii

Ethanolic extract of the leafy stems of *T. vogelii* showed significant acaricidal activity on larvae of *Rhipicephalus sanguineus*. Mortality rates were not adjusted to the Abbott formula (1925) as the average larval mortality in the control lot did not exceed 5%. The larval mortality in the control lots was 3.45%. From six concentrations used, the highest larval mortality was obtained with the highest concentration (20%). The 10%, 5%, 2.5%, 1.25%, and 0.625% induced larval mortalities of 86.36%, 61.97%, 50%, 18.45%, and 31.73%, respectively. The lethal concentration 50 of the ethanolic extract of the leafy stems of *Tephrosia vogelii* was 2.6% (Figure 6). 

The lethal concentrations 10, 50, 90, and 99 and their confidence intervals are shown in Table 5.

**Table 5:** In vitro lethal concentrations of the ethanol extract of T. vogelii

	**Lethal Concentration**	**IC (95%)**
**LC**_10_	0.425	0.137 – 0.755
**LC**_50_	2.600	1.771 – 3.844
**LC**_90_	15.893	8.870 – 50.434
**LC**_99_	69.533	27.132 – 500.043

## Discussion

Tick species, frequencies, and relative abundances

This study revealed a high infestation rate (77.5%) and abundance of ticks in dogs of the Municipality of Abomey-Calavi. This strong presence of ticks could be explained by two factors, which are the season of study and the mode of breeding of dogs examined. The present study was conducted in September and October, two rainy months in Southern Benin. Farougou et al. (2007) ▶ showed that in general, ticks are more present in animals, especially sheeps, during the rainy seasons. The study period was therefore able to influence the rate of infestation. However, the mode of breeding of local breed dogs may also explain the high abundance of ticks. Indeed, the majority of these dogs were almost in constant wandering during the day and much more contact with nature. Moreover, as demonstrated by Smith et al. (2011) ▶, the probability of ticks infestation in this category of dogs is high because they often attend predilection places of ticks as vegetation and crevices.

Several tick species were inventoried in our work. These and other species have been identified in the work of various authors. Four genera of ticks were identified in dogs in Japan by Shimada et al. (2003) ▶. They were Rhipicephalus, Haemaphysalis, Ixodes, and Amblyomma. In this study, *Rhipicephalus sanguineus* was not the most abundant (4.8%) tick while *Haemaphysalis longicornis* was the most abundant (40.3%). However, previous studies in the same country revealed that according to the study area, *Rhipicephalus sanguineus* might be the dominant species (Henna et al., 1990 ▶; Inokuma et al., 1998 ▶) just as it is in the present study. In South Africa, nine species of ticks were counted on the dogs in the Free State Province (Jacobs et al., 2001 ▶). Of these nine species, *Rhipicephalus sanguineus* and *Haemaphysalis leachi* were the most common ticks (73.5% and 22.4%, respectively) and were more abundant compared to this study. In the same study, other species of the genus Rhipicephalus and Amblyomma were inventoried: *Rhipicephalus evertsi evertsi*, *Rhipicephalus follis*, *Rhipicephalus gertrudae*, *Rhipicephalus warbutoni*, and *Amblyomma marmoreum* (Jacobs et al., 2001 ▶). Results of another study in the same country showed the same trend with a preponderance of *Rhipicephalus sanguineus*, *Haemaphysalis leachi*, and *Rhipicephalus simus* (Horak and Matthee, 2003 ▶).

Studies on dogs’ ticks in West Africa are infrequent. In Nigeria, ticks are the most ectoparasites found in dogs with predominance of *Rhipicephalus sanguineus* and *Rhipicephalus longus* (Dipeolu, 1975 ▶; Ugochukwu and Nnadozie, 1985 ▶). A more recent study inventoried *Rhipicephalus sanguineus* and *Ixodes sp* with respective prevalence of 19.2% and 4.5% (Ugbomoiko et al., 2008 ▶).

Many authors have reported *Rhipicephalus sanguineus* as the most common tick in domestic dogs. Nevertheless, in Gabon, Pourrut et al. (2011) ▶ reported that no *Rhipicephalus sanguineus* specimen has been found in dogs examined in some localities and the brown dog tick has been replaced by *Haemaphysalis paraleachi*. This could be explained by several factors, including the area of study, the season, and the randomness of the sampling. In the same study, other ticks of the genus Rhipicephalus among which *Rhipicephalus sulcatus* were identified on domestic dogs.

However, an almost exclusive tropism for the dog, *Rhipicephalus sanguineus* may parasitize other species. Indeed, Farougou et al. (2007) ▶ have reported infestation cases of sheep by the brown dog tick in southern Benin with a relative abundance of 0.76%. It was the same in other studies on ruminants (Jongejan et al., 1987 ▶; François, 2008 ▶). As regards the developmental stages, immature stages (nymphs) of *Rhipicephalus sanguineus* were found in the dogs examined. This is consistent with results of Walker et al. (2007) ▶ who reported that all stages of *R. sanguineus* are found almost exclusively in dogs.

*Haemaphysalis leachi* was found on dogs in the present study which confirms its large distribution and high affinity for dogs. However, it has been found on other hosts carnivores such as red mongoose (*Herpestes sanguinea*) in Gabon (Pourrut et al., 2011 ▶) and even on ruminants, result of close proximity between domestic dogs and livestock (Walker et al., 2007 ▶).

The third genus of ticks, inventoried in this study was Amblyomma. Widespread in Africa, Amblyomma ticks have as usual hosts domestic and wild ruminants such as buffalo, cattle, sheep, and goats. The best known and listed in Africa and Benin is *Amblyomma variegatum*. However, during close proximity with other animals, it readily infests them. This observation was made in our study where it was noted that dogs infested with ticks *Amblyomma variegatum* were those close to goats and sheep. Similarly, Barre (1989) ▶ found this tick, at adult stage, infesting dogs in the West Indies. The large host variability of Amblyomma ticks is therefore confirmed.

**Traditional control methods used against ticks**

Traditional control methods against ticks and other parasites of ruminants (cattle, goats, and sheep) are well documented in Benin (Hounzangbe-Adote, 2001 ▶). Unfortunately, this is not the case with pets such as dogs. This is easily explained by the low priority often given to local breed dogs and little or no economic interest of their breeding in Benin. However, our results showed that dog owners feel concerned by the infestation of their pets by ticks. This is why they use various strategies (manual diptank, seawater, petroleum, motor oil, and vegetable ash). However, it should be noted that, despite these strategies, the infestation rate (77.5%) and the abundance of ticks are high in examined dogs. Moreover, effectiveness of these methods is then relatively weak and uncertain. One of the main reasons is that there is no scientific method in the application of various practices and no study has been done to verify their effectiveness or suggest ameliorations.

**Laboratory culture of Rhipicephalus sanguineus**

In this study, the average preoviposition, oviposition, and incubation durations observed during culture of *Rhipicephalus sanguineus* were 5, 18, and 17 days. These short periods are due to respect of optimal culture conditions of the brown dog tick. They roughly correspond to those reported in the literature and observed in other trials of laboratory culture of *Rhipicephalus sanguineus*.

Indeed, Dantas-Torres (2010) ▶ reported that preoviposition lasts between three days and weeks. The work of Koch (1982) ▶ showed that the duration of oviposition of *Rhipicephalus sanguineus* laboratory with varying conditions (temperature: 10-35 °C, humidity: 15-95%) can last several weeks. Hatching occurs after an incubation period ranging from 6 days to weeks with an average of 30 days (Dantas-Torres, 2008 ▶). The culture trials carried out by (Dantas -Torres, 2008 ▶) showed that at 25 °C, the life cycle of *Rhipicephalus sanguineus* lasts 86-123 days and 65-90 days at 29 °C. Because of its relatively short life cycle, *Rhipicephalus sanguineus* is an ideal tick species for laboratory tests such as efficiency test and experimental infestations. Parameters observed in the present study confirmed that the optimum temperature and humidity for laboratory culture of *Rhipicephalus sanguineus* are 26 °C and 80%, respectively as reported by Dantas-Torres (2010) ▶.

**Phytochemical screening of** ***Tephrosia vogelii***

The phytochemical screening of the leafy stems of *Tephrosia vogelii* revealed the presence of catechol tannins, saponins, reducing compounds, leucoanthocyanins, and sterols-polyterpenes. However, we noted the absence of flavonoids. Nevertheless, rotenone is the main molecule of *Tephrosia vogelii* and its derivatives are flavonoids. This absence can be explained by the existence of several chemotypes of *Tephrosia vogelii* due to variations in soil type, seasons, and growth stage of the plant. Indeed, Noudogbessi et al. (2012) ▶ mentioned the existence of two different chemotypes of *Tephrosia vogelii* in Benin. Furthermore, the qualitative phytochemical screening may not reveal the presence of certain compounds when they are present in very small traces. Yet, as shown by Kalume et al. (2012) ▶, the concentration of rotenone and its derivatives in *Tephrosia vogelii *is low. Indeed, a quantitative study of leafy stems of two chemotypes of *Tephrosia vogelii* revealed rates of 0.044 to 1.13% and 0.014 to 0.66% for rotenone and degueline, respectively (Kalume et al., 2012 ▶). Quantitative studies are often required to ensure the presence or absence of rotenoids in *Tephrosia vogelii* (Freyre and Barnes, 1967 ▶). However, it is noted that these low concentrations are largely sufficient to confer interesting biocidal properties of *Tephrosia vogelii* (Kalume et al., 2012 ▶)

Other authors have made the same observation in their works. Dianzitoukoulou (2008) ▶ found the presence of saponins, polyphenols, and steroids and the absence of alkaloids, terpenoids, and cardiac glycosides. Moreover, he noted the lack of flavonoids in its study samples.

**In vitro acaricidal activity of ethanol extract of leafy stem of** ***Tephrosia vogelii***

Larval mortality obtained in this study was high. It ranged from 18.45 to 100%. The larvicidal potential of ethanolic extract of leafy stem of Rhipicephalus sanguineus is probably due to the chemical composition of *Tephrosia vogelii*. The phytochemical screening performed revealed the presence of catechol tannins, saponins, reducing compounds (sugars), leuco-anthocyanins, and sterols-polyterpenes. Many of these chemical compounds have biocidal properties. In fact, tannins have shown significant acaricide potential on the cattle tick, *Rhipicephalus Boophilus microplus* (Fernández- Salas et al., 2011 ▶). Saponins also exert an inhibitory activity on cold-blooded animals and invertebrates, which was confirmed by Varma and Srivastava (1964). The combined action of these groups of compounds may be responsible for the larvicidal activity observed in vitro.

*Tephrosia vogelii*, commonly known as the “fish bean” or “fish-poison bean” is used by farmers in Africa to control pests on livestock, in cultivated fields as an organic pesticide, and as a medicine for skin diseases and internal worms (PACE, 2013 ▶). Leaf extract of Tephrosia is used as a low cost acaricide in central Kenya and results are encouraging (PACE, 2013 ▶). Because of its interest, several African authors have already evaluated the effect of *Tephrosia vogelii* on various ticks. In these studies, the mortality induced by extracts of *Tephrosia vogelii* was often very high. Kalume et al. (2012) ▶, observed a mortality of 95 and 100% using concentrations of 10 and 20 mg/mL of leaves of two varieties of *Tephrosia vogelii* against Rhipicephalus appendiculatus. The same observation was made when chloroformic, methanolic, aqueous, and etheric extracts of leafy stems of *Tephrosia vogelii* were used on nymphs and adult ticks in Uganda (Matovu and Olila, 2007 ▶). Gadzirayi et al. (2009) ▶ concluded from their work that various concentrations ranging from 50 to 100 g of leaves of *Tephrosia vogelii* in 100 to 200 mL of water could be used to fight against ticks in general by farmers. Similarly, in Benin, Dougnon et al. (2012) ▶ found that the ethanolic extract of the leaves of *Tephrosia vogelii* induced *in vivo* 98.51% mortality in *Amblyomma variegatum*. The authors conclude that the ethanol extract of leaves of *Tephrosia vogelii* can be used as well as Alfapor^®^ in the fight against ticks.

Analysis of results obtained in this study, revealed that:

-Ticks, *Rhipicephalus sanguineus*, *Haemaphysalis leachi*, and *Amblyomma variegatum* infest domestic dogs in the Municipality of Abomey-Calavi.

-*Rhipicephalus sanguineus* is most abundant tick on dogs in this study, however, *Haemaphysalis leachi* is also widespread in domestic dogs.

-Dog owners often develop strategies against the infestation of their animals. It is the use of manual diptank, vegetable ash, petroleum, motor oil, and sea water.

-The ethanolic extract of the leafy stems of *Tephrosia vogelii* has a high acaricidal potential.

- *Tephrosia vogelii* can be used in the fight against ticks of pets and especially dogs.
